# Feeding difficulties in patients with Phenylketonuria

**DOI:** 10.1590/2317-1782/20232021292en

**Published:** 2023-09-25

**Authors:** Alexia Diovana Fernandes da Rocha, Chenia Caldeira Martinez, Lilia Farret Refosco, Tássia Tonon, Ida Vanessa Doederlein Schwartz, Sheila Tamanini de Almeida

**Affiliations:** 1 Universidade Federal de Ciências da Saúde de Porto Alegre - UFCSPA - Porto Alegre (RS), Brasil.; 2 Clínica de Atendimento Psicológico, Faculdade de Fonoaudiologia, Instituto de Psicologia, Universidade Federal do Rio Grande do Sul - UFRGS - Porto Alegre (RS), Brasil.; 3 Serviço de Genética Médica, Hospital de Clínicas de Porto Alegre - HCPA - Porto Alegre (RS), Brasil.; 4 Programa de Pós-graduação em Ciências Médicas, Universidade Federal do Rio Grande do Sul - UFRGS - Porto Alegre (RS), Brasil.; 5 Programa de Pós-graduação em Genética e Biologia Molecular, Universidade Federal do Rio Grande do Sul - UFRGS - Porto Alegre (RS), Brasil.

**Keywords:** Phenylketonurias, Metabolism, Inborn Errors, Feeding Behavior, Food Fussiness, Speech, Language and Hearing Sciences

## Abstract

**Purpose:**

to analyze the results of an instrument that aims to assist in the identification of feeding difficulties in children with Phenylketonuria (PKU), compared to children without the disease.

**Methods:**

cross-sectional, controlled study with a convenience sample composed of patients with PKU and healthy individuals, matched for age and sex. The invitation to participate in the study was made through the dissemination of the research on social networks. The answers were provided by the guardians, 46 controls and 28 patients agreed to participate. In addition to these, 13 guardians of patients being followed up at an Outpatient Clinic for the Treatment of Inborn Errors of Metabolism were invited by phone call, and 12 accepted the invitation. All participants answered the Brazilian Infant Feeding Scale (in Portuguese Escala Brasileira de Alimentação Infantil (EBAI)) electronically.

**Results:**

the study included 86 participants, 40 patients (median of age = 2 years; interquartile range (IQR) = 2 - 4) and 46 controls (median of age = 3.5 years; IQR = 2 - 5.25). Ten (25%) patients and 13 (28.3%) controls had suspicion of feeding difficulties, demonstrating a similar frequency of feeding difficulties between groups. The study found that PKU patients had less feed autonomy (*p* = 0.005), were less breastfed (*p* = 0.002) and used more baby’s bottle than controls (*p* = 0.028).

**Conclusion:**

the frequency of feeding difficulties reported by caregivers was similar between the comparison groups, but children with PKU had less feed autonomy, were less breastfed and used more baby’s bottles when compared to children without the disease.

## INTRODUCTION

Phenylketonuria (PKU) is an inborn error of amino acid metabolism with recessive autosomal inheritance, caused by mutations on the gene that codes the hepatic phenylalanine hydroxylase enzyme (PAH)^(1)^. PAH is responsible for converting the amino acid phenylalanine (Phe) into tyrosine via a tetrahydrobiopterin, molecular oxygen and iron dependent reaction^(2,3)^. A deficiency or absence of PAH activity leads to increased Phe concentrations in the blood to neurotoxic levels^(2,3)^.

Treatment of the disease is based on dietary therapy involving Phe control, requiring reduced natural protein intake and substitution with metabolic formula as a Phe free protein source^(2-4)^. Lack of treatment for the disease can lead to progressive and irreversible neurological damage in patients^(5)^.

PKU prevalence varies considerably between countries and ethnicities^(1-3)^. In Brazil, the estimated prevalence is 1:24780 newborns^(2-6)^. The disease can be detected through early Neonatal Screening, with recommendations that the test be realized between the third and fifth day of life^(1)^. Early intervention, preferably between the seventh and tenth day of life, prevents clinical manifestation of the disease^(1,2)^.

Diet is usually fairly restricted and should be maintained throughout the patient’s life, with regular adjustments made by the medical and nutritional team. The patient can only consume a limited variety of natural foods without restriction, such as some types of fruits, vegetables, fats and sugars^(2-7)^. Feeding plays a fundamental role in infant development and strict dietary therapies such as that for PKU can negatively affect feeding behaviour and development in children^(7)^.

Feeding difficulties are very common in infancy, affecting around 25-35% of normally developing children and 40-80% of children with developmental disorders, independent of sex and socioeconomic factors^(8,9)^. According to Kerzner et al.^(9)^ the ample term “feeding difficulties”, refers to the presence of some type of feeding difficulty in the child, caused by organic or behavioural factors, as well as aspects associated with feeding practices of caregivers^(9)^. As such, it is important to consider the perspective of caregivers when evaluating children.

Recently, the Federal Council of Speech Therapists presented resolution 659, proposing the term “pediatric feeding disorder” (PFD)^(10)^. Additionally, studies and norms have sought to standardize terminology and interventions for feeding difficulties during infancy^(10,11)^. PFD is defined as inhibited oral ingestion that is inadequate for age, and associated organic/medical, nutritional, psychosocial and feeding ability dysfunctions^(11)^. The main factors involved in PFD development can be related to medical conditions, behaviour related to the child-feeding dyad, the feeding environment, and to developmental delays in the child’s feeding abilities, leading to nutritional impairments, due to restrictions in food quantity, quality and/or variety^(11)^.

Feeding difficulties have already previously been reported with inborn errors of metabolism, mainly in PKU^(7,12,13)^. The literature highlights that these patients can be susceptible to developing this condition mainly due to the strictness of dietary therapies^(7,12,13)^. Prior studies have specifically investigated feeding neophobia in this population or evaluated feeding difficulties without a validated instrument. In this context, the present study aimed to analyse the results of an instrument that seeks to aid in identifying feeding difficulties in children with PKU, in comparison with children without the disease, based on reports from caregivers.

## METHODS

The present study is a cross-sectional, controlled study with a convenience sample, approved by the Research Ethics Committee of the Porto Alegre Clinical Hospital (in Portuguese Hospital de Clínicas de Porto Alegre (HCPA)), under process number 2019/0777. The sample was made up of patients with a PKU diagnosis and healthy controls (without PKU or other metabolic diseases), matched for age and sex. The study took place between May and August 2020.

Inclusion criteria for participation in the study were: age between 6 months and 6 years and 11 months. Exclusion criteria for patients and the control were presence of a health condition that could generate bias in the research, such as genetic syndromes, craniofacial malformation, tracheotomy or neurological diseases (in the case of patients, not related directly to damage from PKU). Patients should also be in ongoing dietary treatment with a low Phe diet and use of metabolic formula free of Phe.

Invitation to participate in the study was made by publicizing the research on social media as well as by phone call to patients treated at the Inborn Errors of Metabolism Outpatient Clinic of the HCPA’s Medical Genetics Service. The calls were made by the service’s team. Inclusion in the study was only confirmed after they agreed by signing the Free and Informed Consent (TCLE).

Collected data included: birth date, sex, age at PKU diagnosis, PKU classification, breastfeeding, oral habits, feeding behaviour, gastrointestinal symptoms (nausea, vomiting, diarrhea, stomach pain, gastroesophageal reflux disease (GERD)) and ingestion of metabolic formula, which were obtained from the clinical records. The clinical record used for data collection was developed specifically for this study.

Regarding the PKU classification, the categories “typical” or “mild” were used, as described by Nalin et al.^(14)^. In typical PKU, patients had a plasma Phe concentration at diagnosis greater than 1200 µmol/L and Phe tolerance below 350 mg/day. In mild PKU, the plasma Phe value at diagnosis was from 600-1200 µmol/L and Phe tolerance was greater than 350 mg/day^(14)^.

In terms of the sample subjects, guardians for 46 control subjects and 28 patients agreed to participate in the study. Of the 13 guardians for patients being treated at the HCPA’s Inborn Errors of Metabolism Outpatient Clinic, 12 accepted the invitation. The guardians filled in the online survey using the Google Forms platform. The questionnaire included a clinical record and the Brazilian Infant Feeding Scale (in Portuguese Escala Brasileira de Alimentação Infantil (EBAI))^(15)^.

Identification of feeding difficulties was undertaken using the EBAI, an instrument developed initially in English and French by Ramsay et al.^(16)^, and then translated into Portuguese and validated for use in Brazil by Diniz et al.^(15)^. The scale classifies feeding difficulties according to seriousness and the level of concern of parents/caregivers based on their opinions about the feeding behaviour of their children^(15)^.

The EBAI has 14 items that consider the following aspects: appetite, sensory oral involvement, oral motor development, general parental concern regarding feeding their child, child behaviour at meal times, strategies used by caregivers and reactions of caregivers in relation to feeding their child^(15)^. When responding, caregivers should score each item using a Likert scale (numbers from 1 to 7), marking them according to the degree of intensity of the response. The scale allows us to identify the degree of feeding difficulty, with children presenting scores from 61 to 65 being considered to have mild difficulties, from 66 to 70 as having moderate difficulties and above 70 as having serious difficulties^(15)^.

Statistical analysis was undertaken using the Statistical Package for Social Science program (version 18.0, SPSS Inc., Chicago, IL), with a significance of 5% (*p* ≤ 0.05), with significant results being highlighted with an asterisk on the tables. The categorical variables were summarized using frequency and percentage. The continuous variables were summarized using the median and the interquartile interval (IQR). The statistical Chi-squared, Exact Fisher and Mann-Whitney tests were used for the non-parametric variables.

## RESULTS

Eighty-six participants were included in the study, with 40 patients (median age = 2 years; IQR = 2 - 4; minimum age = 7 months, maximum age = 6 years, 11 months) and 46 controls (median age = 3.5 years; IQR = 2 - 5.25; minimum age = 7 months, maximum age = 6 years, 11 months). The data for sample characterization appear in Table 1.

**Table 1:** Clinical and demographic characteristics of patients with Phenylketonuria and for controls

	Patients (n = 40)	Control (n = 46)	*P*-Value
Sex			0.666
Female	19 (47.5%)	25 (54.3%)	
Male	21 (52.5%)	21 (45.7%)	
Age (years)	2 (2 - 4)	3.5 (2 - 5.25)	0.241
Age at diagnosis (days)	23 (15 - 52)	-	
PKU Classification (n=39)		-	
*Typical*	26 (66.7%)		
*Mild*	13 (33.3%)		
Gastrointestinal symptoms	19 (47.5%)	15 (32.6%)	0.188
Oral habits			
*Bottle*	40 (100%)	40 (87%)	0.028*
Use of bottle (*months*)	29 (18 - 40)	33 (20.25 - 48)	0.915
*Dummy*	24 (60%)	32 (69.6%)	0.374
Use of dummy (months)	31 (22 - 48)	30 (14 - 40.5)	0.606

* Statistical significance by Chi-squared test (*p* ≤ 0.05)

**Caption:** PKU = Phenylketonuria

Thirty patients (75%) and 45 controls (97.8%) were breastfed during some period of their life (*p* = 0.002). In PKU patients, breastfeeding was always associated with metabolic formula free of Phe. In the patient group, five caregivers (12.5%) reported suffering stress when offering metabolic formula to their child. The data related to feeding and feeding difficulties are presented in Table 2.

**Table 2:** Feeding aspects and difficulties in patients with Phenylketonuria and in controls

	Patients (n = 40)	Control (n = 46)	*P*-Value
Breast feeding	30 (75%)	45 (97.8%)	0.002*
Time breastfeeding (months)	5 (3 - 9)	7 (3 - 11.25)	0.631
Feeding behaviours			
*Stress during feeding*	7 (17.5%)	13 (28.3%)	0.309
*Feeding autonomy*	28 (70%)	43 (93.5%)	0.005*
Time feeding			0.213
*<20 min.*	17 (42.5%)	14 (30.4%)	
*20-30 min.*	20 (50%)	23 (50%)	
*>30 min*	3 (7.5%)	9 (19.6%)	
Distractions during meal time			0.329
*TV*	9 (22.5%)	15 (32.6%)	
*Tablet*	1 (2.5%)	0 (0%)	
*Cell phone*	4 (10%)	3 (6.5%)	
*TV, Cell phone*	5 (12.5%)	4 (8.7%)	
*TV, Cell phone and Tablet*	2 (5%)	5 (10.9%)	
*TV, Tablet*	0 (0%)	3 (6.5%)	
*Doesn’t eat with distractions*	19 (47.5%)	16 (34.8%)	
Number of tools for feeding			0.168
*1*	15 (37.5%)	9 (19.6%)	
*2*	10 (25%)	15 (32.6%)	
*3*	12 (30%)	20 (43.5%)	
*4*	1 (2.5%)	2 (4.3%)	
*Doesn’t use*	2 (5%)	0 (0%)	
Feeding difficulties	10 (25%)	13 (28.3%)	0.810

* Statistical significance by Chi-squared test (*p* ≤ 0.05)

According to the EBAI classification, which seeks to assist in evaluating feeding difficulties based on self-reporting from caregivers, it was found that 10 patients (25%) and 13 controls (28.3%) presented results indicative of feeding difficulties, showing a similar frequency in both groups (*p*=0.810). In the patient group, five participants (12.5%) presented criteria for mild feeding difficulty, one (2.5%) for moderate difficulty and four (10%) for serious difficulty. Among the controls, six participants (13%) presented criteria for mild difficulty, four (8.7%) for moderate difficulty and three (6.5%) for serious difficulty. Regarding the total EBAI score, 54 points were observed (IQR = 45.25-60.75) for patients and 50 points (IQR = 46-61) for the controls (*p* = 0.808).

The data for clinical characteristics were analysed and compared with the EBAI score medians (Table 3). The total EBAI score showed a significant relation with the presence of gastrointestinal symptoms (*p* = 0.002), suggesting that those who have gastrointestinal symptoms present higher scores on the scale.

**Table 3:** Comparison between clinical characteristics and total score for the Brazilian Infant Feeding Scale

	Total EBAI score	*P*-Value
Breast feeding		0.969
Yes	53 (45.5 - 61.5)	
No	55.5 (45.25 - 60.25)	
Gastrointestinal symptoms		0.003*
Yes	59 (51 - 63)	
No	49.5 (43 - 56.25)	
PKU classification		0.231
Typical	52 (44.75 - 58)	
Mild	59 (46.5 - 63)	

* Statistical significance according to Mann Whitney test (*p* ≤ 0.05)

**Caption:** EBAI = *Escala Brasileira de Alimentação Infantil* (Brazilian Infant Feeding Scale)

A significant relation between the EBAI result and the presence of gastrointestinal symptoms (*p* = 0.024) was observed. Among the participants with alteration in the result of the scale, 14 presented gastrointestinal symptoms (60.9%).

## DISCUSSION

This was the first study to use the Brazilian Infant Feeding Scale (validated for Brazil) on patients with metabolic disease and feeding restrictions. Our results indicated that the suspicion of feeding difficulties, identified by the reports of caregivers using the scale, was similar in children with and without PKU. Notably, patients presented less autonomy when feeding than the controls, were breastfed less often and used the bottle as a tool for feeding more frequently in comparison with controls.

These results differ from previous studies that showed that mothers of PKU patients noticed more feeding problems in their children than mothers of children free of the disease^(7-9,11-13)^. However, one point to be highlighted is that PKU patients from this study were recruited from spaces where they were periodically monitored by a multidisciplinary team and participated in groups for the exchange of experiences and also to receive guidance about breastfeeding and feeding. This could be a factor that positively altered the outcome for feeding development for these children. It is important to note that it was not possible to carry out a comparison between the prevalence identified in the prior studies with PKU and those of the present study, since different instruments were used to measure the feeding difficulties.

Another relevant variable to be highlighted was “autonomy”, given that it is important for the process of feeding development^(17)^. In terms of the lesser autonomy observed in PKU patients in comparison with the controls, it was believed that this was related to the concern of parents regarding the diet for PKU, which has numerous restrictions, requiring careful control to avoid elevated plasma Phe levels. As such, it is common that guardians prevent children from having easy access to prohibited foods (rich in protein or phenylalanine) and to controlled foods (with some quantity of phenylalanine, but permitted in small portions according to nutritional guidelines). This was observed by a prior study^(18)^, that identified children with PKU, aged between 0 and 2 years, who were spoon fed by their parents for longer and also began eating on their own later than children without the disease.

The lower frequency of breastfeeding in the PKU group (75%) in comparison with the control group (97.8%) was also observed in a study carried out in the United States of America (USA) and Canada, in which the prevalence of breastfeeding after PKU diagnosis was 72.81%^(19)^. Additionally, Kose et al.^(20)^ observed that 61% of children continued being breast fed following the PKU diagnosis, with an average duration of 7.4 ± 4.0 months^(20)^. Notably, breastfeeding should be encouraged in patients and can be successfully incorporated into the PKU diet, as long as children are periodically monitored by a multidisciplinary team, allowing for ongoing dietary adjustments and adequate metabolic control^(21,22)^, since it is known that breastfeeding is one possible factor for prevention of feeding difficulties^(23)^.

An association between indication of feeding difficulties by the EBAI and complaint of gastrointestinal symptoms was also observed, although both groups reported these symptoms (nausea, vomiting, diarrhoea, GERD, constipation, stomach pains). This finding was similar to studies that showed that gastrointestinal symptoms were a risk factor for the development of feeding difficulties in children^(9,11-22,24,25)^. Other studies found that gastrointestinal symptoms were significantly more common in the PKU group than in the control^(7-13)^. However, the study by Evans et al.^(18)^ corroborates the findings from this study, since we also observed that both children with PKU, and those without the disease were similar in the occurrence of gastrointestinal symptoms^(18)^.

The use of a bottle was also found to be more common in the PKU group (100%) in comparison with the control group (87%), a result similar to that observed by Evans et al.^(18)^ and MacDonald et al.^(7)^. The need for daily ingestion of metabolic formula free of Phe leaves these patients more susceptible to using the bottle, a tool that facilitates consumption of metabolic formula. Moreover, although using the bottle is not recommended after the first year of life^(24)^, some mothers use it to give expressed breastmilk to their children^(22)^, since they are able to control the volume and quantity of Phe in this manner.

Other variables such as “time of feeding” or “use of distractions during meal time” were less common in the PKU group in comparison with the control group, while “stress during meal time” was similar between the groups. These outcomes were attributed to parents of patients with diseases that require treatment and regular dietary monitoring, tending to adopt healthy feeding habits for their children, thereby guaranteeing greater metabolic regulation. Furthermore, the study of MacDonald et al.^(13)^ also identified that feeding related stress was similar in children with and without PKU, with parents of patients reporting that the moment of administering metabolic formula to their children was more stressful than feeding them. It was concluded that the support given by the multidisciplinary team, the facilitation of access to information about the disease and sharing of experiences between family members of patients (through mother’s groups, social networks, etc.) can positively influence feeding.

The present study had some limitations, such as the sample size and the impossibility of ascertaining the Phe levels of PKU patients, due to being a study undertaken from afar, without access to medical records. This consequently hampered comparison between feeding difficulties and metabolic control of the disease. Additionally, the investigation of feeding difficulties was carried out from the perspective of caregivers regarding the feeding behaviour of their children, without including a complete clinical assessment. Another point to highlight is that, despite ample publicization on social media, in groups for PKU patient caregivers and through invitation via phone call, there was no way of guaranteeing that bias did not creep into the sample.

For future research, a larger sample size is recommended and the realization of a detailed assessment of the subjects, to obtain more information and confirmation of the findings regarding feeding difficulties in this population. It is necessary to investigate infant feeding in a multi-professional and transdisciplinary manner, to avoid the onset of feeding difficulties. Early guidance regarding feeding is fundamental to construct good feeding habits and for a healthy relationship with feeding.

## CONCLUSION

Based on self-reporting of family members and caregivers this study found that children with PKU tend to present feeding difficulties (25% of the patient sample). However, the findings were similar between the groups with and without PKU. We observed that children with PKU presented less autonomy when feeding themselves, were breastfed less and used a bottle more in comparison with children without the disease. The results also indicated that the gastrointestinal symptoms had a significant association with complaints of feeding difficulties reported by caregivers.
