# Supplementary material for: Feeding difficulties in patients with Phenylketonuria
Source: Codas. 2023 Sep 25;35(6):e20210292. doi: 10.1590/2317-1782/20232021292en (PMC10702708; doi:10.1590/2317-1782/20232021292en)
Supplement: Translation

# Translation: Dificuldades alimentares em pacientes com Fenilcetonúria

**DOI:** 10.1590/2317-1782/20232021292pt

**Authors:** Alexia Diovana Fernandes da Rocha, Chenia Caldeira Martinez, Lilia Farret Refosco, Tássia Tonon, Ida Vanessa Doederlein Schwartz, Sheila Tamanini de Almeida

**Affiliations:** 1 Universidade Federal de Ciências da Saúde de Porto Alegre - UFCSPA - Porto Alegre (RS), Brasil.; 2 Clínica de Atendimento Psicológico, Faculdade de Fonoaudiologia, Instituto de Psicologia, Universidade Federal do Rio Grande do Sul - UFRGS - Porto Alegre (RS), Brasil.; 3 Serviço de Genética Médica, Hospital de Clínicas de Porto Alegre - HCPA - Porto Alegre (RS), Brasil.; 4 Programa de Pós-graduação em Ciências Médicas, Universidade Federal do Rio Grande do Sul - UFRGS - Porto Alegre (RS), Brasil.; 5 Programa de Pós-graduação em Genética e Biologia Molecular, Universidade Federal do Rio Grande do Sul - UFRGS - Porto Alegre (RS), Brasil.

**Keywords:** Fenilcetonúrias, Erros Inatos do Metabolismo, Comportamento Alimentar, Seletividade Alimentar, Fonoaudiologia

## Abstract

**Objetivo:**

analisar os resultados de um instrumento que se propõe a auxiliar na identificação das dificuldades alimentares em crianças com Fenilcetonúria (PKU), em comparação a crianças sem a doença.

**Método:**

estudo transversal, controlado, com amostra de conveniência composta por pacientes com PKU e por indivíduos hígidos, equiparados por idade e sexo. O convite para participação no estudo foi feito por meio de divulgação da pesquisa nas redes sociais. As respostas foram fornecidas pelos responsáveis, sendo que 46 controles e 28 pacientes participaram. Além desses, 13 responsáveis por pacientes em acompanhamento em um Ambulatório de Tratamento de Erros Inatos do Metabolismo foram convidados por ligação telefônica, sendo que 12 aceitaram o convite. Todos os participantes responderam a Escala Brasileira de Alimentação Infantil (EBAI) de forma eletrônica.

**Resultados:**

foram incluídos no estudo 86 participantes, sendo 40 pacientes (mediana de idade, 2 anos; intervalo interquartil (IQR) = 2 - 4) e 46 controles (mediana de idade, 3,5 anos; IQR = 2 - 5,25). Dez (25%) pacientes e 13 (28,3%) controles apresentaram resultados compatíveis com dificuldades alimentares, demonstrando uma frequência semelhante entre os grupos. O estudo observou que os pacientes com PKU apresentaram menos autonomia alimentar (p = 0,005), foram menos amamentados (p = 0,002) e usaram mais mamadeira que os controles (p = 0,028).

**Conclusão:**

a frequência de dificuldades alimentares referidas pelos cuidadores foi semelhante entre os grupos, porém as crianças com PKU demonstraram menos autonomia para se alimentar, foram menos amamentadas e usaram mais mamadeira quando comparadas com as crianças sem a doença.

## INTRODUÇÃO

A Fenilcetonúria (PKU) é um erro inato do metabolismo dos aminoácidos, com herança autossômica recessiva, causada por mutações no gene que codifica a enzima hepática fenilalanina-hidroxilase (PAH)^(1)^. A PAH é responsável por converter o aminoácido fenilalanina (Phe) em tirosina, por meio de reação dependente de tetrahidrobiopterina, oxigênio molecular e ferro^(2,3)^. A deficiência ou a ausência de atividade da PAH acarreta no aumento da concentração de Phe no sangue a níveis neurotóxicos^(2,3)^.

O tratamento da doença é baseado em uma terapia dietética com restrição do aporte de Phe, requerendo uma redução da ingestão de proteínas naturais e a substituição por uma fonte proteica isenta de Phe, a fórmula metabólica^(2-4)^. A falta de tratamento da doença pode resultar em danos neurológicos progressivos e irreversíveis aos pacientes^(5)^.

A prevalência de PKU varia consideravelmente entre países e diferentes etnias^(1-3)^. No Brasil, estima-se uma prevalência de 1:24780 nascidos vivos^(2-6)^. A doença pode ser detectada precocemente na Triagem Neonatal, sendo recomendado que o teste seja feito entre o terceiro e o quinto dia de vida^(1)^. O início precoce do tratamento, preferencialmente entre o 7º e 10º dia de vida, previne as manifestações clínicas da doença^(1,2)^.

A dieta é usualmente bastante restritiva e deve ser mantida por toda a vida, com ajustes feitos regularmente pela equipe médica e nutricional. Apenas uma limitada variedade de alimentos naturais pode ser oferecida ao paciente sem restrições, como alguns tipos de frutas, vegetais, gorduras e açúcares^(2-7)^. A alimentação desempenha um papel fundamental no desenvolvimento infantil e uma dietoterapia rígida como a da PKU pode afetar negativamente o comportamento e o desenvolvimento alimentar da criança^(7)^.

As dificuldades alimentares são muito comuns na infância, afetando cerca de 25-35% das crianças com desenvolvimento normal e 40-80% das crianças com distúrbios do desenvolvimento, independente do sexo e das questões socioeconômicas^(8,9)^. Segundo Kerzner et al.^(9)^, o termo amplo “dificuldades alimentares”, sugere a presença de algum tipo de problema na alimentação da criança, causado por fatores orgânicos ou comportamentais, bem como aspectos ligados às práticas alimentares dos cuidadores^(9)^. E, neste sentido, a opinião dos cuidadores necessita ser considerada como um auxiliar importante no processo de avaliação das crianças.

Recentemente, o Conselho Federal de Fonoaudiologia apresentou a resolução Nº 659, propondo o termo “distúrbio alimentar pediátrico” (DAP)^(10)^, estudos e normativas tem buscado unificar a terminologia e a atuação nos problemas alimentares na infância^(10,11)^. O DAP é definido como ingestão oral prejudicada, que não é apropriada para a idade, sendo associada a disfunção orgânica/médica, nutricional, psicossocial e das habilidades de alimentação^(11)^. Os principais fatores envolvidos no desenvolvimento do DAP podem estar relacionados a condições médicas, ao comportamento da díade alimentador-criança, ao ambiente da alimentação e ao atraso nas habilidades de alimentação da criança, levando a complicações nutricionais, por conta das restrições em quantidade, qualidade e/ou variedade dos alimentos^(11)^.

As dificuldades alimentares já foram previamente descritas nos erros inatos do metabolismo, principalmente na PKU^(7,12,13)^. A literatura aponta que estes pacientes podem estar suscetíveis ao desenvolvimento desta condição, principalmente por conta da rigidez da dietoterapia^(7,12,13)^. Os estudos prévios investigaram especificamente a neofobia alimentar nesta população ou avaliaram as dificuldades alimentares sem um instrumento validado. Neste contexto, o presente estudo tem como objetivo analisar os resultados de um instrumento que se propõe a auxiliar na identificação das dificuldades alimentares em crianças com PKU, em comparação a crianças sem a doença, a partir da opinião dos cuidadores.

## MÉTODO

Trata-se de um estudo transversal, controlado, com amostragem por conveniência, aprovado pelo Comitê de Ética em Pesquisa do Hospital de Clínicas de Porto Alegre (HCPA), sob nº 2019/0777. A amostra foi composta por pacientes com diagnóstico de PKU e controles hígidos (sem PKU e outras doenças metabólicas), equiparados por idade e sexo. O estudo ocorreu entre maio e agosto de 2020.

Os critérios de inclusão para a participação no estudo foram: idade entre 6 meses e 6 anos e 11 meses e a presença de condição de saúde que pudesse gerar viés a pesquisa, como síndrome genética, malformação craniofacial, traqueostomia ou doença neurológica (no caso dos pacientes, não relacionada diretamente às sequelas da PKU), foi considerado critério de exclusão para pacientes e controles. Também, os pacientes deveriam estar em tratamento dietético contínuo com dieta com baixo teor de Phe e uso de fórmula metabólica livre de Phe.

O convite para participação no estudo foi feito por meio de divulgação da pesquisa nas redes sociais e também por ligação telefônica para os pacientes acompanhados no Ambulatório de Tratamento de Erros Inatos do Metabolismo do Serviço de Genética Médica do HCPA, sendo os contatos fornecidos pela equipe do serviço. A inclusão no estudo foi feita somente após eles concordarem com o Termo de Consentimento Livre e Esclarecido (TCLE).

Os dados coletados incluíram: data de nascimento, sexo, idade ao diagnóstico da PKU, classificação da PKU, amamentação, hábitos orais, comportamento alimentar, sintomas gastrointestinais (náusea, vômito, diarreia, constipação, dor de barriga e refluxo gastroesofágico) e ingestão da fórmula metabólica, foram obtidos por meio de ficha clínica. A ficha clínica para preenchimento destes achados foi desenvolvida exclusivamente para o estudo.

Quanto à classificação da PKU, utilizou-se as categorias “clássica” ou “leve”, como descrita por Nalin et al.^(14)^. Na PKU clássica, os pacientes têm concentração plasmática de Phe ao diagnóstico superior a 1200 µmol/L e tolerância à Phe inferior a 350 mg/dia. Na PKU leve, o valor de Phe plasmática ao diagnóstico deve ser de 600-1200 µmol/L e a tolerância à Phe é maior que 350 mg/dia^(14)^.

Sobre os sujeitos da amostra, os responsáveis por 46 controles e por 28 pacientes aceitaram participar do estudo. Dos 13 responsáveis pelos pacientes em acompanhamento no Ambulatório de Tratamento de Erros Inatos do Metabolismo do HCPA, 12 aceitaram o convite. Os responsáveis preencheram um questionário virtual, por meio da plataforma Google Formulários. O questionário incluiu uma ficha clínica e a Escala Brasileira de Alimentação Infantil (EBAI)^(15)^.

A identificação das dificuldades alimentares foi investigada por intermédio da EBAI, um instrumento primeiramente desenvolvido em inglês e francês por Ramsay et al.^(16)^, depois traduzido para o português e validado para uso no Brasil por Diniz et al.^(15)^. A escala classifica as dificuldades alimentares de acordo com a gravidade e o nível de preocupação dos pais/cuidadores a partir da opinião destes sobre o comportamento alimentar de suas crianças^(15)^.

A EBAI é composta por 14 itens que abordam os seguintes temas: apetite, envolvimento sensorial oral, desenvolvimento motor oral, preocupações parentais sobre a alimentação da criança em geral, comportamento da criança na hora da refeição, estratégias usadas pelos cuidadores e reações dos cuidadores em relação à alimentação da criança^(15)^. Ao responder, os cuidadores deveriam classificar cada um dos itens por meio de uma escala de Likert (números de 1 a 7), marcando-os de acordo com o grau de intensidade das respostas. A escala possibilita a detecção do grau da dificuldade alimentar, sendo que as crianças que apresentam pontuações de 61 a 65 são consideradas com dificuldades leves, de 66 a 70 com dificuldades moderadas, e acima de 70 com dificuldades graves^(15)^.

A análise estatística foi realizada utilizando o programa Statistical Package for Social Sciences (versão 18.0, SPSS Inc., Chicago, IL), com valor de significância de 5% (p ≤ 0,05), sendo que os resultados significativos foram destacados com asterisco nas tabelas. As variáveis categóricas foram sumarizadas por meio de frequência e de porcentagem. As variáveis contínuas foram sumarizadas por meio de mediana e intervalo interquartil (IQR). Os testes estatísticos Qui-Quadrado, Exato de Fisher e Mann-Whitney foram usados nas variáveis não paramétricas.

## RESULTADOS

Oitenta e seis participantes foram incluídos neste estudo, sendo 40 pacientes (mediana de idade, 2 anos; IQR = 2 - 4; idade mínima = 7 meses, idade máxima = 6 anos) e 46 controles (mediana de idade, 3,5 anos; IQR = 2 - 5,25; idade mínima = 7 meses, idade máxima = 6 anos). Os dados de caracterização da amostra estão na Tabela 1.

**Tabela 1:** Características clínicas e demográficas dos pacientes com Fenilcetonúria e dos controles

	**Pacientes (n = 40)**	**Controles (n = 46)**	**Valor de p**
Sexo			0,666
Feminino	19 (47,5%)	25 (54,3%)	
Masculino	21 (52,5%)	21 (45,7%)	
Idade (anos)	2 (2 - 4)	3,5 (2 - 5,25)	0,241
Idade ao diagnóstico (dias)	23 (15 - 52)	-	
Classificação da PKU (n=39)		-	
*Clássica*	26 (66,7%)		
*Leve*	13 (33,3%)		
Sintomas gastrointestinais	19 (47,5%)	15 (32,6%)	0,188
Hábitos orais			
*Mamadeira*	40 (100%)	40 (87%)	0,028*
Uso de *mamadeira* (*meses*)	29 (18 - 40)	33 (20,25 - 48)	0,915
*Chupeta*	24 (60%)	32 (69,6%)	0,374
Uso de chupeta (meses)	31 (22 - 48)	30 (14 - 40,5)	0,606

* Significância estatística pelo Teste Qui-quadrado (p ≤ 0,05)

**Legenda:** PKU = Fenilcetonúria

Trinta pacientes (75%) e 45 controles (97,8%) foram amamentados em algum período de suas vidas (p = 0,002). Nos pacientes com PKU, a amamentação foi sempre associada à fórmula metabólica livre em Phe. No grupo de pacientes, 5 cuidadores (12,5%) referiram ser estressante o momento de oferecimento da fórmula metabólica ao seu filho. Os dados referentes à alimentação e às dificuldades alimentares estão dispostos na Tabela 2.

**Tabela 2:** Aspectos da alimentação e dificuldades alimentares em pacientes com Fenilcetonúria e em controles

	**Pacientes (n = 40)**	**Controles (n = 46)**	**Valor de p**
Amamentação	30 (75%)	45 (97,8%)	0,002*
Tempo de amamentação (meses)	5 (3 - 9)	7 (3 - 11,25)	0,631
Comportamentos alimentares			
*Estresse na alimentação*	7 (17,5%)	13 (28,3%)	0,309
*Autonomia alimentar*	28 (70%)	43 (93,5%)	0,005*
Tempo de alimentação			0,213
*<20 min.*	17 (42,5%)	14 (30,4%)	
*20-30 min.*	20 (50%)	23 (50%)	
*>30 min*	3 (7,5%)	9 (19,6%)	
Distrações durante a refeição			0,329
*TV*	9 (22,5%)	15 (32,6%)	
*Tablet*	1 (2,5%)	0 (0%)	
*Celular*	4 (10%)	3 (6,5%)	
*TV, Celular*	5 (12,5%)	4 (8,7%)	
*TV, Celular e Tablet*	2 (5%)	5 (10,9%)	
*TV, Tablet*	0 (0%)	3 (6,5%)	
*Não come com distrações*	19 (47,5%)	16 (34,8%)	
Quantidade de utensílios na alimentação			0,168
*1*	15 (37,5%)	9 (19,6%)	
*2*	10 (25%)	15 (32,6%)	
*3*	12 (30%)	20 (43,5%)	
*4*	1 (2,5%)	2 (4,3%)	
*Não usa*	2 (5%)	0 (0%)	
Dificuldades alimentares	10 (25%)	13 (28,3%)	0,810

* Significância estatística pelo Teste Qui-quadrado (p ≤ 0,05)

De acordo com a classificação da EBAI, que se propõe a auxiliar na avaliação das dificuldades alimentares a partir do autorrelato dos cuidadores, obteve-se que: 10 pacientes (25%) e 13 controles (28,3%) apresentaram resultados indicativos de dificuldades alimentares, mostrando frequência semelhante em ambos os grupos (p = 0,810). No grupo dos pacientes, cinco (12,5%) apresentaram critério para dificuldade alimentar leve, um (2,5%) para dificuldade moderada e quatro (10%) para dificuldade grave. Dentre os controles, seis (13%) apresentaram critério para dificuldade leve, quatro (8,7%) para dificuldade moderada e três (6,5%) para dificuldade grave. Sobre o escore total da EBAI, observou-se 54 pontos (IQR = 45,25 - 60,75) para os pacientes e de 50 pontos (IQR = 46 - 61) para os controles (p = 0,808).

Os dados referentes às características clínicas foram analisados e comparados com as medianas dos escores da EBAI (Tabela 3). O escore total da EBAI teve relação significativa com a presença de sintomas gastrointestinais (p = 0,002), sugerindo que aqueles que possuíam sintomas gastrointestinais apresentaram escores maiores na escala.

**Tabela 3:** Comparação entre características clínicas e escore total da Escala Brasileira de Alimentação Infantil

	Escore total	Valor de p
	**EBAI**	
*Amamentação*		0,969
*Sim*	*53 (45,5 - 61,5)*	
*Não*	*55,5 (45,25 - 60,25)*	
Sintomas gastrointestinais		0,003*
Sim	59 (51 - 63)	
Não	49,5 (43 - 56,25)	
Classificação da PKU		0,231
Clássica	52 (44,75 - 58)	
Leve	59 (46,5 - 63)	

* Significância estatística pelo Teste Mann Whitney (p ≤ 0,05)

**Legenda:** EBAI = Escala Brasileira de Alimentação Infantil

Observou-se relação significativa entre o resultado da EBAI e a presença de sintomas gastrointestinais (p = 0,024). Entre os sujeitos com alteração no resultado da escala, 14 apresentaram sintomas gastrointestinais (60,9%).

## DISCUSSÃO

Este foi o primeiro estudo a utilizar a Escala Brasileira de Alimentação Infantil (validada no Brasil) em pacientes com doença metabólica e restrição alimentar. Nossos resultados indicaram que a suspeita de dificuldades alimentares, identificada pelo relato dos cuidadores à escala, foi semelhante em crianças com e sem PKU. Salientamos que os pacientes apresentaram menos autonomia na alimentação do que os controles, foram menos amamentados e utilizaram a mamadeira como utensílio para alimentação com maior frequência em comparação aos controles.

Estes resultados diferem dos estudos prévios, que demonstram que as mães de pacientes com PKU percebem mais problemas alimentares em seus filhos do que as mães de crianças sem a doença^(7-9,11-13)^. No entanto, um ponto a ser destacado é que os pacientes com PKU, deste estudo, foram recrutados de espaços onde são acompanhados periodicamente por uma equipe transdisciplinar, participavam de grupos para troca de experiências e também recebiam orientações sobre amamentação e alimentação. Este pode ser um fator que modificou positivamente o desfecho do desenvolvimento alimentar destas crianças. É importante ressaltar que não foi possível realizar uma comparação entre a prevalência identificada em estudos prévios com PKU e a do presente estudo, pois foram utilizados instrumentos diferentes para mensurar as dificuldades alimentares.

Outra variável relevante de ser destacada foi a “autonomia”, uma vez que ela é importante no processo de desenvolvimento alimentar^(17)^. Em relação a menor autonomia identificada nos pacientes com PKU em comparação aos controles, entende-se que pode estar relacionada à preocupação dos pais a respeito da dieta para PKU, que possui várias restrições, necessitando ser controlada cuidadosamente para evitar níveis altos de Phe no sangue. Ou seja, é comum que os responsáveis evitem que as crianças tenham livre acesso aos alimentos proibidos (ricos em proteína ou fenilalanina) ou aos alimentos controlados (com alguma quantidade de fenilalanina, mas permitidos em pequenas porções conforme a orientação nutricional). Este achado foi observado por um estudo prévio^(18)^, que identificou crianças com PKU, de idade entre 0 e 2 anos, que foram alimentadas com colher pelos pais por mais tempo e também começaram a comer sozinhas mais tarde do que crianças sem a doença.

A menor frequência de amamentação observada no grupo PKU (75%) em comparação ao grupo controle (97,8%) também já foi descrita em um estudo realizado nos Estados Unidos e no Canadá, em que a prevalência de amamentação após o diagnóstico de PKU foi de 72,81%^(19)^. Ainda, Kose et al.^(20)^ observaram que 61% das crianças continuaram sendo amamentadas após o diagnóstico de PKU, com média de duração de 7,4 ± 4,0 meses^(20)^. Destaca-se que a amamentação deve ser incentivada nos pacientes e pode ser incorporada com sucesso à dieta da PKU, desde que esses sejam acompanhados periodicamente por uma equipe multiprofissional, possibilitando ajustes dietéticos constantes e controle metabólico adequado^(21,22)^, pois sabe-se que a amamentação é um possível fator de proteção para as dificuldades alimentares^(23)^.

Ainda, foi encontrada associação entre a indicação de dificuldades alimentares apontada pela EBAI e a queixa de sintomas gastrointestinais, embora ambos os grupos tenham referido estes sintomas (náuseas, vômitos, diarreia, refluxo gastroesofágico, constipação, dor de barriga). Este achado é semelhante a estudos que mostram que os sintomas gastrointestinais seriam um fator de risco para o desenvolvimento de dificuldades alimentares em crianças^(9,11-22,24,25)^. Outros estudos observaram que os sintomas gastrointestinais eram significativamente mais comuns no grupo com PKU do que no grupo controle^(7-13)^. Entretanto, o estudo de Evans et al.^(18)^ corrobora com os achados identificados no presente trabalho, pois também observaram que tanto as crianças com PKU, quanto as crianças sem a doença, apresentavam semelhança na ocorrência de sintomas gastrointestinais^(18)^.

Também foi observado que o uso de mamadeira foi mais frequente no grupo PKU (100%) quando comparado ao grupo controle (87%), resultado semelhante ao identificado por Evans et al.^(18)^ e por MacDonald et al.^(7)^. A necessidade da ingestão diária de fórmula metabólica isenta em Phe torna esses pacientes suscetíveis ao uso da mamadeira, utensílio que facilita a administração da fórmula metabólica. Além disso, embora não seja recomendado após o primeiro ano de vida^(24)^, sabe-se que algumas mães utilizam a mamadeira para oferecer o leite materno ordenhado aos seus filhos^(22)^, pois assim é possível controlar o volume e a quantidade de Phe.

Outras variáveis como “tempo de duração da alimentação” ou “uso de distrações durante a refeição” foram menos autorreferidas no grupo PKU em comparação aos controles, ao passo que “estresse durante as refeições” foi semelhante entre os grupos. Atribui-se estes resultados ao fato de que pais de pacientes com doenças que necessitam de tratamento e acompanhamento dietético regular, tendem a oferecer hábitos alimentares saudáveis a seus filhos, visando menor descontrole metabólico. Além disso, o estudo de MacDonald et al.^(13)^ também identificou que o estresse relacionado à alimentação foi semelhante em crianças com e sem PKU, sendo que os pais dos pacientes comentaram que o momento de administrar fórmula metabólica aos seus filhos era mais estressante do que alimentá-los. Conclui-se que o suporte da equipe multiprofissional, a facilidade do acesso às informações sobre a doença e o compartilhamento de experiências entre os familiares de pacientes (por meio de grupos de mães, das redes sociais, etc.) podem influenciar positivamente na alimentação.

O presente estudo apresentou algumas limitações, como o tamanho amostral e a impossibilidade de coletar os níveis de Phe dos pacientes com PKU, por ter sido um estudo feito à distância, sem acesso aos prontuários, impedindo a comparação entre dificuldades alimentares e controle metabólico da doença. Além disso, a investigação das dificuldades alimentares foi feita por meio da percepção dos cuidadores a respeito do comportamento alimentar dos seus filhos, sem incluir uma avaliação clínica completa. Outro ponto de destaque é que, apesar da ampla divulgação nas redes sociais, nos grupos de cuidadores de pacientes com PKU e nos convites por ligação telefônica, não há como garantir que não tenha ocorrido viés de seleção nesta amostra.

Para pesquisas futuras, recomenda-se um tamanho amostral maior e a realização de uma avaliação detalhada dos sujeitos, para obtenção de mais informações e confirmação dos achados das dificuldades alimentares nesta população. É importante que a alimentação infantil seja trabalhada de forma multiprofissional e transdisciplinar, como forma de evitar o surgimento das dificuldades alimentares. As orientações precoces a respeito da alimentação são fundamentais para a construção de bons hábitos alimentares e de uma relação saudável com o alimento.

## CONCLUSÃO

Este estudo observou que as crianças com PKU estão propensas a apresentarem dificuldades alimentares, a partir do autorrelato dos familiares e cuidadores (25% de frequência na amostra de pacientes). Contudo, os achados foram semelhantes entre os grupos com e sem PKU. Identificou-se que as crianças com PKU possuem menos autonomia para se alimentar, são menos amamentadas e usam mais mamadeira quando comparadas às crianças sem a doença. Os resultados encontrados também indicaram que os sintomas gastrointestinais tiveram relação significativa com as queixas de dificuldades alimentares referidas pelos cuidadores.
